# Sexual Dimorphism of the Lateral Angle of the Petrous Bone in Children: Growth Patterns and the Influence of Cranial Width

**DOI:** 10.3390/biology14060628

**Published:** 2025-05-29

**Authors:** Lukas Waltenberger, Stefan Lettner, Anton Dobsak, Martin Dockner, Lena Hirtler, Stefan Tangl

**Affiliations:** 1 Institute for Prehistory and Historical Archaeology, University of Vienna, Franz-Klein-Gasse 1, 1190 Vienna, Austria; lukas.waltenberger@univie.ac.at; 2 Austrian Archaeological Institute, Austrian Academy of Sciences, Dominikanerbastei 16, 1010 Vienna, Austria; 3 Core Facility Hard Tissue and Biomaterial Research, Karl Donath Laboratory, Medical University of Vienna, Sensengasse 2, 1090 Vienna, Austria; stefan.lettner@meduniwien.ac.at (S.L.); anton.dobsak@meduniwien.ac.at (A.D.); 4 Austrian Cluster for Tissue Regeneration, 1200 Vienna, Austria; 5 Department of Anthropology and Core Facility for Micro-Computed Tomography, University of Vienna, Djerassiplatz 1, 1030 Vienna, Austria; martin.dockner@univie.ac.at; 6 Division of Anatomy, Center for Anatomy and Cell Biology, Medical University of Vienna, Währinger Straße 13, 1090 Vienna, Austria; lena.hirtler@meduniwien.ac.at

**Keywords:** internal acoustic meatus, biauricular width, sexual dimorphism, cranial base

## Abstract

Background: The lateral angle of the internal acoustic meatus of the petrous bone is a sexually dimorphic feature used for sex determination, particularly in fragmented or cremated remains. However, studies show conflicting results regarding its accuracy, and the reasons for its dimorphism remain unclear. The aim of this study is to analyze sexual dimorphism in subadult individuals and to examine the association of the lateral angle with cranial breadth as an explanation for its sexual dimorphism, as well as interpopulation differences. Methods: We measured the lateral angle and biauricular breadth in 204 individuals (birth to 30 years) using CT scans from Austrian 19th-century anatomical collections and data from the New Mexico Decedent Image Database. Results: This study revealed that the sexual dimorphism of the lateral angle and cranial dimensions manifests during puberty, along with a strong association between the lateral angle and the biauricular breadth. Additionally, this study noted interpopulation variability in cranial breadth, with different levels of sexual dimorphism observed across diverse populations. Conclusions: The findings offer a potential explanation for the observed variability in lateral angle measurements across studies and the limitations of universal cut-off points as a sex-determination method in osteology.

## Simple Summary

The lateral angle of the internal ear canal opening in the skull is sometimes used to determine the sex of human remains in biological anthropology, especially when the skeleton is damaged or burned. However, earlier studies have shown mixed results regarding the reliability of this method. This study looked at subadults to investigate if this feature is already different between boys and girls before adulthood and whether its size is related to the width of the skull. We measured the lateral angle in 204 individuals, from newborns up to 30 years old, using detailed 3D scans of 19th-century Austrian skulls. Because there were few older teenagers in this collection, additional data from modern forensic CT scans (NM, USA) were used. The results showed that the difference between males and females in the lateral angle does not appear until puberty. It was also found that the width of the skull is closely linked to the lateral angle measurements. Differences between populations were also observed. These findings help explain why earlier studies sometimes disagreed and suggest that using a single cut-off value to determine sex based on this angle may not work well for all groups of people.

## 1. Introduction

The determination of sex in human skeletal remains is typically performed macroscopically, with the morphologic assessment of pelvic and cranial traits being the primary method of analysis [1,2]. In well-preserved skeletons, this approach is reliable, with accuracies ranging from 90 to 97%, depending on the methods and traits utilized [3,4,5]. However, in forensic cases and archaeological studies, the preservation of the bones is frequently poor and highly fragmented, necessitating the use of less reliable but nevertheless sexually dimorphic features. Such methods commonly employ length measurements of sexually dimorphic anatomical structures [6,7]. This is due to the general assumption that males are, on average, larger than females and that skeletal structures are also more robust because of the higher muscle mass in males [6]. A notable skeletal element that is frequently recovered in inhumation burials and often survives even a cremation process in at least large pieces is the petrous bone [8,9,10].

The petrous bone, a part of the temporal bone, is a robust and dense skeletal element located at the cranial base, which is a relatively well-protected area of the cranium. The internal acoustic meatus is the conduit through which the facial nerve, vestibulocochlear nerve, and two blood vessels (the arteria and vena labyrinthi) pass to supply the inner ear [11]. Originating in the cerebellum and the truncus encephali, both nerves are of particular interest in this anatomical region. Anatomically, the internal acoustic meatus forms a conically shaped canal that becomes narrower from medial to lateral. The facial nerve exits the petrous bone via the facial canal, while the vestibulocochlear nerve supplies the inner ear [11,12].

One feature that has been shown to be sexually dimorphic is the so-called lateral angle of the internal acoustic meatus, first defined by Wahl [13]. The lateral angle is the acute angle formed by the lateral wall of the internal acoustic meatus and the posterior surface of the petrous bone [13,14]. A number of studies have indicated that this angle may be used for sex determination in inhumation burials [9,10,14,15] and in cremations [13,16,17]. The lateral angle is generally larger in females than in males [9,13,14,15,18,19,20,21]. According to Wahl [13], a lateral angle above 45° is indicative of the subject being female, whereas a lateral angle measuring 45° is indicative of the subject being male. While several studies confirmed the 45° cut-off point with good accuracy [9,15,22], others have proposed divergent cut-off points that have proven more effective for their particular population-specific samples [16,19,23]. Furthermore, several studies have indicated that the lateral angle is an imprecise and unreliable method because of its high degree of overlap between males and females and large confidence regions [13,14,17,18,20,21,24]. The accuracy of using the lateral angle for sex determination exhibited significant variation across studies Table 1), ranging from an accuracy comparable to a coin toss, approximately 50% [18,21], to over 80% of individuals being correctly classified [9,19]. Thus, researchers hypothesized an association of the lateral angle with age [10,15,16,20,22,25], and this association was suspected to be population-specific [23,26]. Furthermore, the techniques utilized to measure the lateral angle, particularly those techniques employed for the measurements of virtual scans and models or casts derived from dry bone material, appear to yield divergent results [18,23,24].

It is important to note that the petrous bone reaches approximately half of its full size at two years of age and does not undergo further bone remodeling of the otic capsule, the bone surrounding the inner ear, and proceeds with much slower rates of development until adulthood [30,31]. Consequently, researchers have assumed that the lateral angle is already sexually dimorphic in subadult individuals, likely attributable to increased rugosity of muscle attachment at the temporal and occipital bones in males [9,20]. This assumption is further supported by the study by Duquesnel Mana, et al. [32], who analyzed the basicranial shape and found that the sexual dimorphism is most pronounced in the region of the sigmoid and transverse sulci, although these differences were not significant. Studies have indicated that the sexual dimorphism in cranial shape is predominantly caused by size differences and variation in the robustness of muscle attachment sites, with male crania being larger and more rugged on average [32,33]. Thus, one can hypothesize that the sexual dimorphism of the lateral angle may evolve during puberty. However, studies conducted on subadult samples are rare, and the findings are inconsistent. For instance, Gonçalves, Campanacho and Cardoso [20] claimed that the lateral angle is already sexually dimorphic in children younger than 15 years, with the strongest results observed in the 2- to 5-year age group. However, the sample size of this study was small (*n* = 35), and when the age group “2- to 5-year” was excluded from the prediction, the accuracy dropped from 63 to 56%. Thus, these results can be attributed to the small and uneven sample size. In contrast, Afacan, Onal, Akansel and Arslan [25] failed to identify any statistically significant differences. A slight decrease in the lateral angle during childhood was found in both studies.

The aim of this study was to analyze the development of sexual dimorphism of the lateral angle from birth up to adulthood in a sample with known sex and age at death. In addition to the repeated testing of eligibility using the lateral method as a method for sex determination, it is imperative to delve deeper into the underlying reasons why a tiny aperture at the cranial base exhibits sexual dimorphism and the manner in which it evolves during childhood. The hypothesis is that sexual dimorphism may develop during puberty or, alternatively, in early adulthood until complete skeletal maturation. This comprehensive approach could facilitate a more profound understanding of the lateral angle geometry and its interpopulation variation.

## 2. Methods

**Figure 1:**
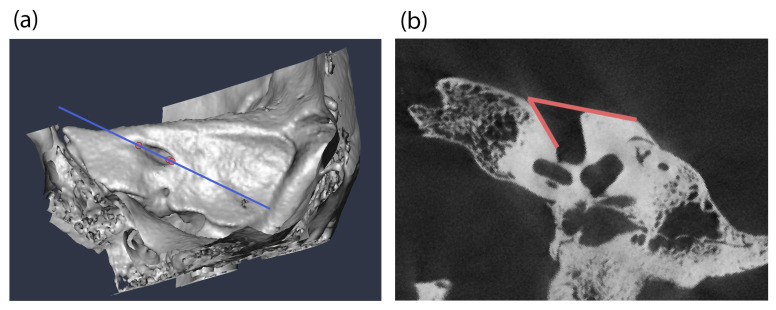
Overview of the lateral angle measurement procedure. (**a**) The intersection of the internal acoustic meatus in the midsagittal plane using the landmark-based method on CT scans. Landmarks are depicted as red dots. (**b**) Measurement of the lateral angle in the CT scan slide aligned to the midsagittal plane of the internal acoustic meatus.

We used four samples in this study to analyze the development of the lateral angle during childhood, with the objective of identifying causal factors. Initially, the micro-CT scans of three museum osteological collections from 19th-century Austria were examined: 50 skulls from the Collection of the Anatomical Institute of Vienna (Vienna, Austria [34]), 49 from the Collection of the Anatomical Institute of Graz (with only inscriptions on the crania available; Graz, Austria), and 17 from the Terzer Collection (Vienna, Austria [35,36]). These three collections primarily comprise skulls of subadult individuals. Some background information, including sex, age at death, and year of death, is known, mostly from inscriptions on the skulls themselves. The Terzer Collection predominantly contains samples of individuals under three years of age, the Collection of the Anatomical Institute of Vienna contains subjects under 10 years of age, and the Collection of the Anatomical Institute of Graz spans the age range from three years to 25 years. Exclusion criteria for all samples were pathological conditions affecting the cranial shape and any known or visible cranial trauma on the CT scans. Inclusion criteria were crania of individuals who died between birth and 30 years of age. As male features at the crania tend to become further pronounced in early adulthood, we expand our sample to individuals who died in their 20s. All crania were micro-CT scanned at the Core Facility of Microcomputed Tomography of the Department of Evolutionary Anthropology, University of Vienna (scanner model Viscom X8060, Viscom SE, Hannover, Germany) with an isovoxel size of 39–79 µm depending on the field of view. The micro-CT scans were semiautomatically segmented in the software Amira 6.1.1 [37]. The internal acoustic meatus was then intersected using the landmark-based method developed by Waltenberger, Heimel, Skerjanz, Tangl, Verdianu and Rebay-Salisbury [23]. Angle measurements of the lateral angle were performed on images using the Fiji 1.54g software [38] and the angle measurement tool (Figure 1).

Furthermore, we measured the bi-auricular breadth (AUB, Martin [39]) in order to ascertain a potential association with the lateral angle. Given the rarity of individuals between the ages 17 to 25 in the historical samples (5 individuals), which were exclusively males, the decision was made to enhance the dataset by including recent data from individuals who died between the 12th and 30th year of life. This approach was taken to identify any potential changes in cranial breadth from puberty to early adulthood. The data were obtained from the New Mexico Decedent Database (NMDID, Alburquerque, NM, USA [40]) by selecting groups of equal size for males and females and including four individuals of the same age to ensure continuous data from 12 to 30 years (*n* = 114). The study exclusively included individuals of European ancestry, thereby facilitating comparability with the Austrian historical collections. The NMDID collection contains whole-body CT scans of corpses examined in the coroner’s office in Albuquerque with a resolution of 0.5 × 0.5 mm per pixel and a slice thickness of 0.6 mm. Given the lower resolution of the NMDID database in comparison to that of the micro-CT scans, we decided to only measure the bi-auricular width as a proxy for lateral angle changes, thus avoiding a methodological bias in the lateral angle measurements. The subsequent analysis of the data was conducted using multiple linear regression models, binary logistic regression, LOESS regression, mixed regression models to account for samples from different collections, and response operating characteristic (ROC) curves in R statistics 4.4.1 utilizing the packages visreg [41], ggplot2 [42], boot [43,44], and verification [45]. In the multiple regression models, the lateral angle was used as the independent variable, whereas predictors were the biauricular breadth and age. In the binary logistic regression models, we predicted sex with the lateral angle and biauricular breadth, respectively. To test for differences in the development of the biauricular width in females and males, we analyzed differences in the smoothed moving average of the LOESS regressions, including a bootstrap analysis. For this, 1000 new samples were drawn based on the original data, with cases retained after each draw. The LOESS regressions were calculated, and differences between the prediction of the age were noted. Based on these differences, bias-corrected and accelerated confidence intervals were calculated. If the differences between males and females were larger than 95% prediction intervals, these time periods were determined as showing significantly different development rates of the biauricular width. A level of significance of *p* = 0.05 was used, and all significant *p*-values were corrected using the Bonferroni–Holm correction [46] to deal with multiple testing.

## 3. Results

First, the subjects from the Grazer collection who were older than 21 years were excluded from this study. The reason for the exclusion was that these subjects formed a distinct cluster in the scatterplots, deviating from the primary point cloud. Furthermore, these subjects demonstrated higher measurements of the bi-auricular breadth in comparison to the NMDID data. As the inclusion criteria for these individuals in the Grazer collection were unknown, and they may have been selected for anomalies, they were excluded from further study. During the course of data collection, 19 individuals (12 males and 6 females) from the NMDID sample were excluded due to the absence of cranial CT scans or the presence of visible antemortem and perimortem trauma of the cranium that was not disclosed in the background information. A descriptive overview of the four collections can be seen in Table 2; raw data are presented in Supporting Information Table S1.

**Figure 2:**
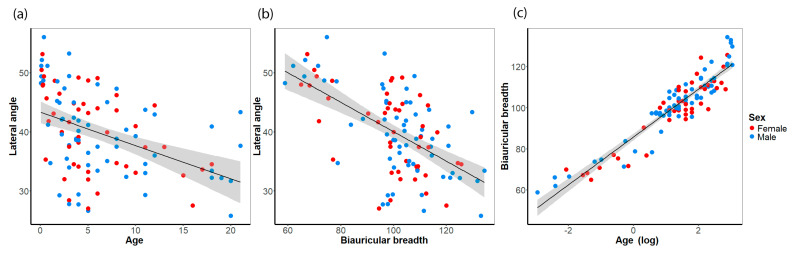
Scatter plots showing the association of analyzed variables. (**a**) association of the lateral angle and age, (**b**) association of the lateral angle and the biauricular breadth, and (**c**) association of the biauricular breadth with the log-transformed age.

First, we used mixed regression to account for samples from different collections but decided to use simpler regression models because the sample distributions were in line with other studies (e.g., Afacan, Onal, Akansel and Arslan [25]). We analyzed the developmental trajectory of the lateral angle during the childhood period. To this end, a linear regression model was employed, incorporating the lateral angle as a function of age, with both female and male subjects combined. The linear regression model revealed a significant decrease in the lateral angle in subadults (*R*^2^ = 0.188, *p* < 0.001, *β_angle_* = −0.335, Figure 2a). The highest values of the lateral angle were measured in children in their first year of life (mean = 48.1°, sd = 5.1°). In a similar manner, the biauricular breadth showed a significant association with the lateral angle (*R*^2^ = 0.336, *p* < 0.001, *β_angle_* = −1.349, Figure 2b). The biauricular breadth accounted for a considerably higher portion of the total variance in the lateral angle than age. By employing a regression analysis, in which age (log-transformed) and the biauricular breadth were dependent and independent variables, respectively, it was demonstrated that age influences both the lateral angle and the biauricular breadth (Figure 2c). As anticipated, multicollinearity may underlie the observed association between the lateral angle and age, given that children are undergoing growth, which leads to an increase in cranial dimensions with age. To address this potential multicollinearity, we opted to calculate a third linear regression model incorporating cranial breadth as a covariate to adjust for age. This analysis revealed that the association between age and the lateral angle became non-significant, although the partial regression coefficient of the biauricular breadth remained significant (adjusted *R*^2^ = 0.325, *p* < 0.001, *β_age_* = 0.092, *p_β-age_* = 0.595, *β_AUB_* = −0.274, *p_β-AUB_* < 0.001). This finding implies that cranial breadth is the fundamental factor that influences the size of the lateral angle.

**Figure 3:**
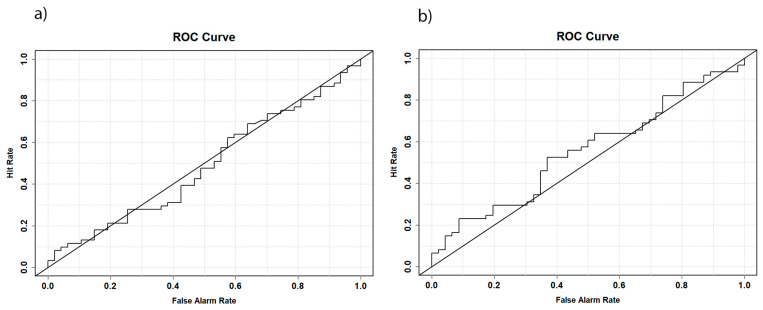
ROC curves showing the quality of the classification models to predict the sex based on (**a**) the lateral angle and (**b**) the biauricular breadth.

Next, we examined sex differences in the lateral angle during the developmental stage of childhood. In the event of a significant difference in the lateral angle already present in children, a good classification could be expected using a ROC model. However, the classification of the lateral angle using a ROC curve (*AUC* = 0.488, Figure 3a) did not follow this expectation. The model performed no better than chance, suggesting that the lateral angle cannot be used for sex determination in subadult individuals. A similar outcome was observed when the biauricular width was analyzed (*AUC* = 0.553, Figure 3b). These findings imply that the lateral angle is predominantly influenced by the cranial dimensions. Individuals with a larger cranial breadth exhibited smaller lateral angle measurements and vice versa. Furthermore, no sexual dimorphism of the lateral angle could be detected in the subadult samples. Next, we analyzed the biauricular breadth in detail as a proxy for the lateral angle in different samples to bring further insights into this hypothesis.

**Figure 4:**
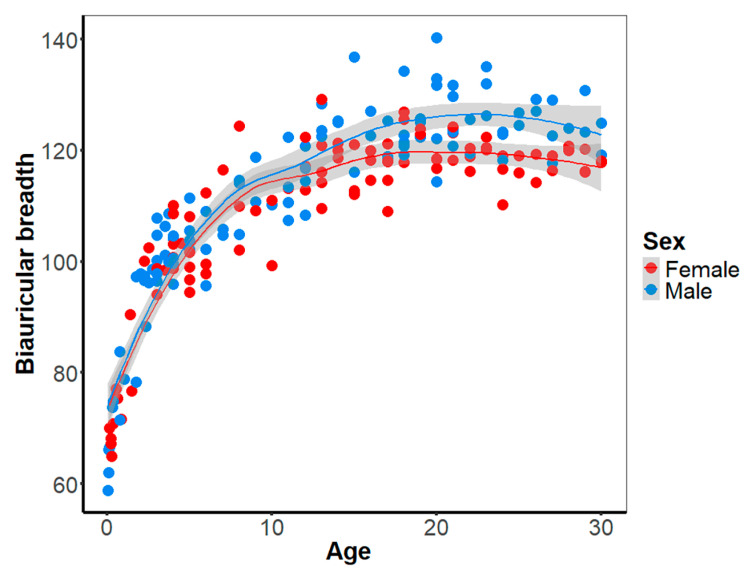
Development of the sexual dimorphism of the biauricular breadth as a proxy for the development of the lateral angle. The LOESS regression lines of females (red) and males (blue) show a similar development in children. From 12 years onwards, the biauricular breadth increases much stronger in males than in females, and from 16 years onwards, the 95% confidence regions of both sexes separate, suggesting a sufficiently large sexual dimorphism that could be detected by statistical methods.

The development of the biauricular breadth over the course of childhood is comparable between females and males. In both males and females, the cranial breadth undergoes a logarithmic development, marked by a substantial increase in the initial years of life and subsequent decline from the 10th year onwards. The onset of sexual dimorphism has been observed to occur during puberty (Figure 4, Supporting Information Figure S1), as evidenced by the separation of the LOESS regression lines between females and males at the age of 16 years. This separation suggests that from this point onward, sexual dimorphism may be sufficiently large to be discernible through statistical methods. To evaluate this assumption in detail, we compared the slopes of the non-linear regression between males and females. Cranial breadth exhibited a comparable development and accelerated growth in individuals up to the ninth year of life. From the age of ten years onwards, the cranial breadth development is higher in males than in females. Statistical analysis reveals that the slopes of these two phases, the resting period in females and the rapid development in males during puberty, are significantly different. The utilization of bootstrap resampling to minimize noise in the results substantiates this finding. The results show that sexual dimorphism of the cranial breadth, and consequently the lateral angle, is established during puberty (see Supporting Information Figure S1 for further details). The negative values indicate that the male specimens exhibit a greater biauricular breadth than the female specimens.

## 4. Discussion

This study has shown that the sexual dimorphism in the lateral angle is not present before puberty, suggesting that it develops during pubertal growth. The lateral angle is strongly associated with the biauricular breadth. Many cranial dimensions, including the cranial breadth, are known to be sexually dimorphic in many populations and may explain differences in the accuracy of the use of the lateral angle as a method of sex determination in osteology. Variation in the cut-off points of the lateral angle is likely to be influenced by interpopulation differences.

### 4.1. The Development of the Lateral Angle During Childhood

This study was unable to identify a sexual dimorphism of the lateral angle in children. As expected, the most plausible explanation would be that the sexual dimorphism of the lateral angle would form during puberty, as for most sexually dimorphic skeletal features. However, it provides significant insights into its development and potential causes of sexual dimorphism in the lateral angle. The present results are consistent with those of previous studies that also failed to identify significant differences in the lateral angle in subadults [25,47], yet they stand in contrast to the results reported by Gonçalves, Campanacho and Cardoso [20]. The divergent outcomes are hypothesized to be predominantly influenced by the limited sample size and the heterogeneous composition of the age groups in the study. The present study found that the lateral angle is, on average, the highest around birth and decreases during childhood in both sexes. This finding suggests that the sexual dimorphism of the lateral angle is established during puberty or early adulthood and is predominantly influenced by the sexual dimorphism of cranial dimensions. The sexual dimorphism of the lateral angle may not solely reflect overall cranial size; it may instead be the result of local anatomical constraints. The spatial configuration of the cranial nerves and blood vessels near the internal acoustic meatus, for example, might allow for a more linear neurovascular pathway in larger crania, thereby influencing the angular morphology. This suggests a functional component beyond mere size scaling. An analysis of the development of the lateral angle during childhood reveals a notable heterogeneity in our sample, ranging from 11 to 16 years of age with a limited number of cases. This may have a bearing on the results, especially with regard to the risk of false-negative results for sexual dimorphism of the lateral angle. Furthermore, the changes observed in the lateral angle may not be adequately represented by a linear regression model; alternatively, a non-linear model, such as a LOESS function, may prove more suitable. The examination of the development of the lateral angle in the age group of two to 16 years revealed that the negative association of the lateral angle with age completely vanished. This finding indicates that the most substantial alterations in the lateral angle primarily occur during the first years of life, coinciding with the period of accelerated growth of the petrous bone and subsequently during the adolescent phase. However, this trend is similar in both sexes, suggesting that the sexual dimorphism of the lateral angle will not already be evident in small children but will likely form as a kind of fine adaptation later in development.

The association between the lateral angle and cranial breadth, despite its simplified nature, offers intriguing insights into the development of the lateral angle and its sexual dimorphism and may offer a rationale for the inconsistent results reported by various researchers and potentially explain inter-population variability. A parallel can be drawn between the development of the lateral angle and that of the biauricular breadth in infants and toddlers, which gradually slows down over time, mirroring the development of the lateral angle. The present dataset was augmented with data from the NMDID, which yielded further insights into the developmental trajectories of cranial breadth until the 30th year of life. The results indicate that the development is predominantly different in males and females from 10 to 17 years of age, suggesting that this is the time period in which the sexual dimorphism will form. While acknowledging the limitations of the biauricular breadth as an estimate for the lateral angle, the results may offer a potential explanation for the observed variability in the accuracy of cut-off points for sex determination by lateral angle measurement. This variability has been documented in several studies (e.g., Masotti, Succi-Leonelli and Gualdi-Russo [24], Gonçalves, Thompson and Cunha [27]), while others have reported significant differences between both sexes and sex-determination methods with good accuracy (e.g., Norén, Lynnerup, Czarnetzki and Graw [9], El-sherbeney, Ahmed and Ewis [19], Waltenberger, Heimel, Skerjanz, Tangl, Verdianu and Rebay-Salisbury [23]).

Cranial dimensions demonstrate significant variability between populations, including the presence of sexual dimorphism at the cranium [48,49,50]. The present study evaluated the biauricular breadth in the NMDID sample (*n* = 96) as well as the Howell dataset (*n* = 2524, Howells [51], Howells [52], Howells [53]). Both datasets showed a significant association between the biauricular breadth and sex in binary logistic regression models (NMDID: *p* < 0.001, *R*^2^ = 0.260, Howell’s dataset: *p* < 0.001, all populations: *R*^2^ = 0.171, modern European populations: *R*^2^ = 0.298). This finding indicates that the lateral angle is strongly influenced by interpopulation differences. Controversially, in scenarios where a pronounced sexual dimorphism is observed in cranial dimensions, the lateral angle emerges as a reliable sex-determination method, exhibiting a high degree of accuracy. Conversely, the lateral angle can be utilized as a sex-determination method with high accuracy if a pronounced sexual dimorphism is present in cranial dimensions. Consequently, it is not possible to define one universal cut-off point as a sex-determination method. The development of methods for sex determination necessitates adaptation and validation across diverse populations to ensure its efficacy. Furthermore, it is challenging to apply cut-off points developed using recent data to archaeological remains. This is particularly evident in the case of prehistoric European populations, which often exhibit a greater degree of robustness compared to modern humans. Previous publications, for instance, from Austria, have demonstrated highly significant differences in the biauricular breadth in prehistoric grave fields from Austria (e.g., Berner [54]). Similarly, we could define cut-off points for sex determination for Austrian Late Bronze Age populations with good accuracy [23], although the sample size was quite small (*n* = 35). Given the reduced sexual dimorphism of the cranium observed in contemporary populations, the sample size may be a contributing factor to the failure of the lateral angle to yield significant results in the aforementioned studies. The presence of subtle sex differences in the lateral angle may have been obscured by random variation in the limited datasets employed in these studies. The identification of such differences necessitates the presence of substantial variances within small samples to detect more modest variations, a strategy that was frequently impracticable in studies of the lateral angle due to a multitude of factors.

### 4.2. Reproducibility of Studies

Furthermore, different cut-off points and the heterogeneity of published data on the lateral angle suggest a further issue: A review of publications reveals a range of lateral angle size measured from less than 30° to up to 80° in adults (e.g., Wahl [13], Masotti, Succi-Leonelli and Gualdi-Russo [24]). Given the proximity of the lateral angle to brain structures, it is implausible that this variation merely represents biological variation. We suspect that the lateral angle measurements are highly dependent on the methods used, which also reduces replicability between studies. This is particularly evident in older studies, such as those by Wahl [13] and Norén, Lynnerup, Czarnetzki and Graw [9], which utilized casts of the internal acoustic meatus made from clay or silicone. Such casting materials have been demonstrated to potentially compromise the integrity of the original bones due to adhesion issues, in addition to the occurrence of shrinkage effects in the casts, which further complicates the accuracy of the measurements. Consequently, researchers adapted the original methods to 3D imaging techniques and employed CT scans of petrous bones. However, it should be noted that clinical CT scans generally provide a maximal resolution of 0.5 × 0.5 × 0.6 mm per voxel, which is inadequate for precise measurement of the lateral angle. Consequently, micro-CT scanning provides high-resolution images, which reduces the random variability in the measurements and should be the method of choice. In addition, the application of the original method on virtual data is intuitive. We propose the utilization of the landmark-based method described in Waltenberger, Heimel, Skerjanz, Tangl, Verdianu and Rebay-Salisbury [23], which has demonstrated reduced inter- and intra-observer error in comparison to manual alignment techniques and may offer enhanced replicability over previous methodologies.

### 4.3. Limitations of This Study

There are several limitations to this study. First, our sample may be biased. We do not know the inclusion criteria used to select individuals for the historical anatomical collections. The researchers may have included the remains randomly, depending on who died in the area and who was available and permitted to be collected. Likely, the historical sample may be comparative to frail individuals of an archaeological context and may not represent a healthy population. Some individuals were excluded in advance because of visible developmental conditions that may affect the shape of the cranium and, therefore, the lateral angle (e.g., premature suture closure). Evidence that our sample may represent a living population can be found in the study by Afacan, Onal, Akansel and Arslan [25]. They measured the lateral angle in 58 subadult patients aged between one month and 17 years. Their results showed a similar sample distribution across childhood, with greater variability in newborns and less variability in children.

Secondly, data on the lateral angle were only available for subadults, with a substantial proportion derived from individuals who died prior to puberty. Therefore, the results of the changes in the lateral angle in adolescents have to be treated with caution. Using cranial width as a proxy for changes in the lateral angle is only model of approximation (one-third of the variation in the lateral angle can be explained by the biauricular breadth; 66% remains unknown). In this study, we used the most likely association with cranial breadth as a proxy because the lateral angle is part of the petrous bones, which are predominantly located in the mediolateral direction. However, causal factors are rarely simple, and we can expect at least a weak association with cranial length, too.

Overall, the results of the lateral angle are consistent with the results of the biauricular breadth. However, the study needs to be repeated in the future with direct data on the lateral angle of individuals from puberty to young adulthood to better understand its development and the cause of sexual dimorphism. We suggest testing its causal factors first on a population that already shows a large sexual dimorphism of cranial dimensions, as in these samples, the association with causal factors is likely to be larger and not so easily hidden by random noise in the dataset. Finally, we measured the lateral angle on CT scans using the method published by Waltenberger, Heimel, Skerjanz, Tangl, Verdianu and Rebay-Salisbury [23]. As lateral angle measurements appear to be highly dependent on the method used, we would like to emphasize that this study may not be comparable with other studies on this topic due to methodological issues.

## 5. Conclusions

This study shows that the sexual dimorphism of the lateral angle probably developed during puberty, as did most other anatomical structures exhibiting sex differences. Therefore, the lateral angle cannot be reliably used for sex determination in subadult individuals. Furthermore, we found a significant association of the lateral angle with the biauricular breadth as an underlying factor of sexual dimorphism, as well as an association with age. Cranial width is a sexually dimorphic dimension with different variability between populations. This association indicates interpopulation differences and probably explains why the lateral angle has been published as sexually dimorphic in some studies while others have failed to find a significant association. In addition, the results may explain why universal cut-off points are insufficient and why customized cut-off points need to be developed for different populations.

## Figures and Tables

**Table 1:** Summary of lateral angle measurements in males and females from different studies. Large variability can be observed due to differences between the populations and the different methods used.

**Population**	**Period**	**Method**	***n***	**Lateral Angle Measurement [°]**				**Accuracy for Sex Determination [%]**	**Study**
				**Females**		**Males**			
				**Mean**	**sd**	**Mean**	**sd**		
Germany	archaeological (last 1200 years)	cast	70	68	10	54	9	-	Wahl [13]
Germany	modern	cast	269	57	12	46	11	67.4	Graw, Wahl and Ahlbrecht [14]
Germany and Sweden (combined)	modern archaeological (late Viking age to early medieval)	cast	133	48	7	39	6	82.8	Norén, Lynnerup, Czarnetzki and Graw [9]
Turkey	modern	CT	92	46	7	42	7	-	Akansel, Inan, Kurtas, Sarisoy, Arslan and Demirci [15]
Egypt	modern	CT	120	50	6	42	6	80.8	El-sherbeney, Ahmed and Ewis [19]
Italy	modern (cremated)	cast	160	35	9	32	6	58.1	Masotti, Succi-Leonelli and Gualdi-Russo [24]
Denmark	modern	CT	77	47	7	43	8	62.3	Morgan, Lynnerup and Hoppa [21]
Portuguese	modern (cremated)	cast	54	50	13	50	17	-	Gonçalves, et al. [27]
Greek	modern	CT	102	42	5	41	5	53.4	Bonczarowska, McWhirter and Kranioti [18]
Italy	modern	CT	100	44	10	40	9	-	Gibelli, et al. [28]
Brazil	modern	CT	150	46	8	40	9	62.7	Pezo-Lanfranco and Haetinger [26]
Poland	Bronze Age to Iron Age (cremated)	CT	6	49	1	33	6	-	Hałuszko and Guziński [29]
Austria	Late Bronze Age	CT	25	38	5	28	2	80.0	Waltenberger, Heimel, Skerjanz, Tangl, Verdianu and Rebay-Salisbury [23]

**Table 2:** Sample composition of the four collections (Collection of the Anatomical Institute of Vienna, Collection of the Anatomical Institute of Graz, Terzer Collection, and NMDID). Adult individuals over 21 years of the Graz collection were excluded beforehand.

Sample Composition							
Age							
	Sex	*n*	Mean	sd	Median	Min	Max
Vienna	male	30	3.8	2.6	3.3	0.1	11.0
	female	20	4.6	3.3	4.0	0.1	17.0
Graz	male	23	11.7	6.2	11.0	3.0	21.0
	female	18	9.3	4.3	8.5	3.0	18.0
Terzer	male	8	2.0	3.7	0.8	0.1	11.0
	female	9	0.7	0.5	0.5	0.2	1.5
NMDID	male	45	20.8	5.3	21.0	12.0	30.0
	female	51	20.9	5.5	21.0	12.0	30.0
**Lateral angle**							
Vienna	male	30	40.3	7.1	39.9	26.7	53.3
	female	20	41.5	6.0	42.4	31.9	50.5
Graz	male	23	37.1	5.7	37.5	25.8	48.0
	female	18	35.4	5.7	34.6	27.0	46.2
Terzer	male	8	45.1	9.2	48.6	29.3	56.0
	female	9	45.9	5.2	47.8	35.3	53.2
**Biauricular breadth**							
Vienna	male	30	98.2	13.0	100.0	58.9	114.1
	female	20	100.7	9.4	100.0	70.0	116.6
Graz	male	23	114.8	11.5	114.5	95.7	134.4
	female	18	108.9	8.8	109.8	94.4	125.6
Terzer	male	8	79.2	14.2	76.6	62.0	110.7
	female	9	73.6	7.6	71.7	65.0	90.4
NMDID	male	45	124.5	5.4	123.6	114.3	140.3
	female	51	118.9	3.4	118.6	110.3	129.2

## Data Availability

The data that support the findings of this study are available in Supporting Information Table S1 and Supporting Information Figure S1.

## Supplementary Materials

The following supporting information can be downloaded at https://www.mdpi.com/article/10.3390/biology14060628/s1, Supporting Information Figure S1: Deviations of the LOESS regression lines between females and males during development; Supporting Information Table S1: Data used in this study.
