# Supplementary material for: Sexual Dimorphism of the Lateral Angle of the Petrous Bone in Children: Growth Patterns and the Influence of Cranial Width
Source: Biology (Basel). 2025 May 29;14(6):628. doi: 10.3390/biology14060628 (PMC12189165; doi:10.3390/biology14060628)
Supplement: Supplementary file 1 [file biology-14-00628-s001.zip › Supporting Information Figure S1.pdf]

Supporting Information Figure S1

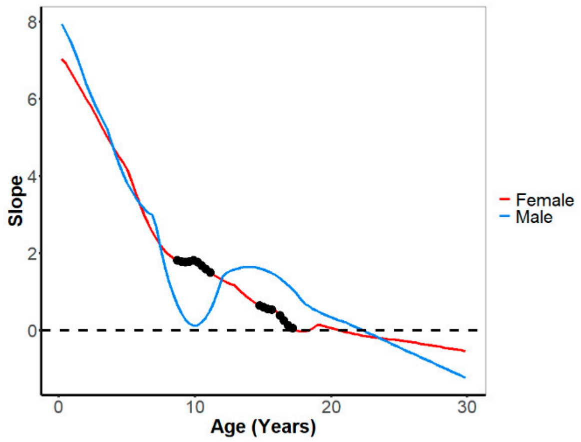

This diagram shows deviations of the LOESS regression lines between females and males during development. Statistically significant deviations of the slopes occur in the age range of 9 to 18 years. This suggests that the sexual dimorphism predominantly form during puberty.
