# Supplementary material for: Sexual Dimorphism of the Lateral Angle of the Petrous Bone in Children: Growth Patterns and the Influence of Cranial Width
Source: Biology (Basel). 2025 May 29;14(6):628. doi: 10.3390/biology14060628 (PMC12189165; doi:10.3390/biology14060628)
Supplement: Supplementary file 1 [file biology-14-00628-s001.zip › Supporting Information Table S1.pdf]

Supporting Information Table S1

Data used in this study.

Collection	ID	Age at death	Sex	Lateral angle	AUB
Vienna	120.009/646	0.1	Male	48.2	58.9
Vienna	120.012/649	0.1	Male	49.4	66.1
Vienna	120.014/651	0.1	Female	50.5	70
Vienna	120.015/652	0.1	Male	52.2	66.5
Vienna	120.035/672	2	Male	44.9	97.2
Vienna	120.038/675	2.2	Male	42.1	96.6
Vienna	120.039/676	2.2	Female	40	100
Vienna	120.040/677	2.2	Male	39.6	97.4
Vienna	120.041/678	2.5	Female	32	102.5
Vienna	120.045/682	2.8	Male	35.5	98.6
Vienna	120.047/684	3	Female	41.7	94.1
Vienna	120.048/685	3	Male	53.2	96.5
Vienna	120.049/686	3	Male	34	100.2
Vienna	120.051/688	3	Male	37.7	104.7
Vienna	120.054/691	3.5	Female	44.8	98.5
Vienna	120.057/694	3.8	Male	38.8	108.6
Vienna	120.060/697	4	Female	38.2	98.7
Vienna	120.061/698	4	Female	39.1	110.2
Vienna	120.064/701	4	Male	40.2	104.1
Vienna	120.066/703	5	Female	43.2	96.7
Vienna	120.067/704	5	Female	31.9	108.1
Vienna	120.068/705	5	Male	36.5	102.2
Vienna	120.069/706	5	Male	34.4	105.5
Vienna	120.079/716	8	Male	37.5	114.1
Vienna	120.080/717	10	Male	39.3	110.3
Vienna	120.083/879	1.8	Male	45.2	97.3
Vienna	120.084/880	2.5	Male	47.4	96.2
Vienna	120.090/886	4.5	Female	44.7	103.3
Vienna	120.101/969	2.3	Male	42.2	88.4
Vienna	120.103/971	5	Male	41.2	103.9
Vienna	120.109/986	2	Male	29.3	97.8
Vienna	120.115/992	3.5	Male	45	106.4
Vienna	120.118/995	5	Male	26.7	111.4
Vienna	120.119/996	6	Female	49.1	99.5
Vienna	120.120/997	7	Female	40	116.6
Vienna	122.032/749	17	Female	33.6	109
Vienna	122.096/819	11	Male	34.6	107.5
Vienna	122.106/829	3.5	Male	42.4	101.1
Vienna	125.002/1063	3.8	Male	38.6	99.9
Vienna	125.206/1008	2	Female	46.5	97.4
Vienna	125.605/1181	3	Male	49.5	107.8
Vienna	125-030/1138	4	Male	29.4	99.7
Vienna	126.302/925	4	Female	43.8	100.7
Vienna	126.303/926	4	Female	49.2	103.2
Vienna	126.320/1094	4	Female	34.1	108.7
Vienna	127.001/1146	4	Male	27.7	95.9
Vienna	127.608/634	8	Male	47.3	104.9
Vienna	300.706/612	6	Female	44	97.8
Vienna	300.813/629	5	Female	48.7	99
Graz	51	12	Female	44.5	112.9
Graz	94	23	Male	31	157.6
Graz	126	25	Male	41.8	161.7
Graz	144	21	Male	37.7	120.8
Graz	150	24	Male	44	158
Graz	176	23	Male	35.3	158.2
Graz	185	20	Male	31.7	131.7
Graz	193	20	Male	25.8	133
Graz	219	18	Male	40.9	120.8
Graz	222	21	Male	43.3	129.8
Graz	230	19	Male	32.2	125
Graz	235	18	Male	33.4	134.3
Graz	236	23	Male	43	161.6
Graz	356	3	Female	28.4	98.8
Graz	360	3	Female	37.5	98.8
Graz	363	4	Male	38.6	100.6
Graz	366	4	Male	41.9	104.7
Graz	380	5	Female	27	94.4

Graz	383	5	Female	33.2	101.8
Graz	385	6	Male	37.5	102.3
Graz	386	6	Male	33.4	109
Graz	388	6	Male	48	95.7
Graz	391	6	Female	29.6	112.3
Graz	394	7	Female	35.1	104.8
Graz	395	7	Male	35.1	105.9
Graz	396	7	Male	45	104.8
Graz	402	8	Female	43.6	102.1
Graz	403	8	Male	38.8	114.6
Graz	404	8	Female	46.2	110
Graz	407	9	Male	40.4	110.7
Graz	408	9	Female	34.2	109.2
Graz	410	9	Male	32.4	118.7
Graz	412	10	Female	41	111
Graz	413	10	Female	33.1	99.3
Graz	415	11	Female	37.4	113.2
Graz	417	12	Male	43	108.3
Graz	418	11	Male	43.7	113.4
Graz	423	13	Female	37.4	109.6
Graz	430	15	Female	32.7	112.1
Graz	461	25	Male	34.9	168.3
Graz	463	18	Female	34.5	125.6
Graz	471	22	Male	34.4	164.7
Graz	484	18	Male	32.3	121.4
Graz	487	24	Male	27.8	151.3
Graz	598	12	Male	36	114.5
Graz	609	11	Male	33	122.3
Graz	657	16	Female	27.5	120
Graz	685	8	Female	34.8	124.3
Graz	687	3	Male	27.8	97.8
Terzer	ZE-000301	0.2	Female	47.8	68.2
Terzer	ZE-000302	0.3	Female	48	65
Terzer	ZE-000304	0.3	Male	48.7	73.8
Terzer	ZE-000305	0.3	Female	49.4	70.8
Terzer	ZE-000308	0.6	Female	45.7	75.3
Terzer	ZE-000310	0.4	Male	56	74.8
Terzer	ZE-000312	0.8	Female	41.8	71.7
Terzer	ZE-000314	1	Male	34.7	78.8
Terzer	ZE-000316	1.4	Female	43.1	90.4
Terzer	ZE-000317	1.5	Female	48.6	76.7
Terzer	ZE-000318	0.8	Male	41.2	83.8
Terzer	ZE-000320	0.5	Female	35.3	77.1
Terzer	ZE-000322	0.1	Male	51.2	62
Terzer	ZE-000328	0.2	Female	53.2	67.3
Terzer	ZE-000329	0.7	Male	51.2	71.4
Terzer	ZE-000334	1.8	Male	48.5	78.3
Terzer	ZE-000348	11	Male	29.3	110.7
NMDID	100169	20	Male	NA	140.3
NMDID	100195	17	Female	NA	114.6
NMDID	100749	23	Male	NA	132.1
NMDID	101245	27	Male	NA	117.7
NMDID	101377	24	Female	NA	119
NMDID	101496	22	Male	NA	125.6
NMDID	101510	26	Male	NA	127.1
NMDID	101569	29	Male	NA	130.8
NMDID	101581	20	Male	NA	114.3
NMDID	101797	17	Female	NA	118
NMDID	102148	18	Female	NA	126.9
NMDID	102325	25	Male	NA	126.8
NMDID	102384	24	Male	NA	122.9
NMDID	102526	30	Male	NA	119.2
NMDID	102557	21	Female	NA	123.2
NMDID	102716	26	Male	NA	129.2
NMDID	103495	22	Female	NA	119
NMDID	103968	20	Male	NA	122.1
NMDID	104011	14	Female	NA	120
NMDID	104228	25	Male	NA	124.5
NMDID	104440	20	Female	NA	116.8
NMDID	105007	13	Female	NA	116.1
NMDID	105323	27	Male	NA	122.7
NMDID	105378	12	Female	NA	117.2
NMDID	105577	28	Male	NA	124

NMDID	105875	23	Male	NA	126.2
NMDID	106172	21	Male	NA	123.3
NMDID	106232	22	Male	NA	119.5
NMDID	106578	12	Male	NA	116.8
NMDID	106677	15	Female	NA	121
NMDID	107186	29	Female	NA	116.2
NMDID	107935	19	Male	NA	125.6
NMDID	108247	24	Male	NA	123.3
NMDID	108300	21	Male	NA	123.6
NMDID	108487	29	Male	NA	123.3
NMDID	108698	28	Female	NA	120
NMDID	109288	26	Female	NA	114.3
NMDID	109357	27	Female	NA	116.3
NMDID	109404	15	Male	NA	136.8
NMDID	109939	28	Female	NA	120.3
NMDID	110723	21	Male	NA	131.8
NMDID	110943	24	Male	NA	118.3
NMDID	111138	26	Female	NA	119.3
NMDID	111780	16	Male	NA	127
NMDID	113033	19	Male	NA	122.3
NMDID	114236	21	Female	NA	118.2
NMDID	114394	27	Male	NA	129.1
NMDID	115034	20	Female	NA	118.4
NMDID	116034	13	Female	NA	120.9
NMDID	116346	24	Female	NA	116.6
NMDID	116536	23	Female	NA	120.1
NMDID	118091	23	Male	NA	135
NMDID	118155	18	Male	NA	122.8
NMDID	118210	17	Male	NA	118.9
NMDID	118570	23	Female	NA	122.3
NMDID	118674	30	Male	NA	125
NMDID	119082	15	Male	NA	116.2
NMDID	119356	17	Male	NA	125.3
NMDID	122413	28	Female	NA	120.8
NMDID	123088	27	Female	NA	116.4
NMDID	123112	17	Female	NA	121.2
NMDID	123570	17	Male	NA	118.8
NMDID	123618	14	Female	NA	118.6
NMDID	125003	15	Female	NA	112.8
NMDID	126383	18	Female	NA	117.9
NMDID	126882	29	Female	NA	116.1
NMDID	128907	19	Male	NA	125.7
NMDID	129054	17	Female	NA	118.2
NMDID	130211	25	Female	NA	116
NMDID	132080	20	Female	NA	118.5
NMDID	132750	16	Male	NA	122.7
NMDID	134893	13	Male	NA	128.4
NMDID	136279	24	Female	NA	110.3
NMDID	137088	13	Male	NA	122.6
NMDID	137318	30	Female	NA	118.1
NMDID	139126	27	Female	NA	119.1
NMDID	141547	12	Male	NA	120.8
NMDID	143868	13	Female	NA	114.3
NMDID	145340	13	Male	NA	123.5
NMDID	146044	29	Female	NA	120.3
NMDID	148810	18	Male	NA	119.2
NMDID	150487	21	Female	NA	124.2
NMDID	153801	19	Female	NA	123.8
NMDID	153893	14	Male	NA	125.4
NMDID	155748	12	Female	NA	122.3
NMDID	165233	30	Female	NA	117.9
NMDID	168962	14	Male	NA	125.1
NMDID	169082	22	Female	NA	120.3
NMDID	182887	14	Female	NA	121.3
NMDID	185974	22	Female	NA	116.3
NMDID	190366	25	Female	NA	119
NMDID	190979	19	Female	NA	122.9
NMDID	193516	13	Female	NA	129.2
NMDID	195202	16	Female	NA	118.3
NMDID	198374	16	Female	NA	114.6
NMDID	199792	23	Female	NA	120.6
